# Phylogenetic transmission clusters among newly diagnosed antiretroviral drug-naïve patients with human immunodeficiency virus-1 in Korea: A study from 1999 to 2012

**DOI:** 10.1371/journal.pone.0217817

**Published:** 2019-06-05

**Authors:** Yoon-Seok Chung, Ju-Yeon Choi, Myoung-Su Yoo, Jae Hyun Seong, Byeong-Sun Choi, Chun Kang

**Affiliations:** 1 Division of Viral Diseases, Center for Laboratory Control and Infectious Diseases, Korea Centers for Disease Control and Prevention, Cheongju, Republic of Korea; 2 Division of Viral Diseases Research, Center for Research of Infectious Diseases, National Institute of Health, Korea Centers for Disease Control and Prevention, Cheongju, Republic of Korea; Hebei Provincial Center for Disease Control and Prevention, CHINA

## Abstract

Population-level phylogenetic patterns reflect both transmission dynamics and genetic changes, which accumulate because of selection or drift. In this study, we determined whether a longitudinally sampled dataset derived from human immunodeficiency virus (HIV)-1-infected individuals over a 14-year period (1999–2012) could shed light on the transmission processes involved in the initiation of the HIV-1 epidemic in Korea. In total, 927 sequences were acquired from 1999 to 2012; each sequence was acquired from an individual patient who had not received treatment. Sequences were used for drug resistance and phylogenetic analyses. Phylogenetic and other analyses were conducted using MEGA version 6.06 based on the GTR G+I parameter model and SAS. Of the 927 samples, 863 (93.1%) were classified as subtype B and 64 were classified as other subtypes. Phylogenetic analysis demonstrated that 104 of 927 patient samples (11.2%) were grouped into 37 clusters. Being part of a transmission cluster was significantly associated with subtype-B viruses, infection via sexual contact, and the infection of young males. Of all clusters, three (~8.1%) that comprised 10 individual samples (22.2% of 45 individuals) included at least one member with total transmitted drug resistance (TDR). In summary, HIV transmission cluster analyses can integrate laboratory data with behavioral data to enable the identification of key transmission patterns to develop tailored interventions aimed at interrupting transmission chains.

## Introduction

The extremely high diversity of human immunodeficiency virus (HIV) has been attributed to its high replication capacity and the high frequency of errors introduced by reverse transcriptase during replication. HIV-1 is the most common virus types worldwide and has been classified into four groups as follows: M (major), N (non-M, non-O), O (outer), and P (pending the identification of further human cases); group P was identified recently in two Cameroonian patients. HIV-1 group M can be further classified into nine subtypes including A–D, F–H, and K [1]. This extensive diversity has led to frequent recombination between strains, resulting in several circulating recombinant forms (CRFs) and a very high number of unique recombinant forms (URFs) [2–5]. To date, 72 CRFs have been isolated, and this number is expected to increase in the future [6].

The unequal distribution of different HIV-1 genotypes worldwide results from the global transmission and spread of certain variants or the limited spread of local endemic strains [1]. Subtype B is predominant in the Americas, Western Europe, and Australia [7–9], whereas subtype B is also the most abundant genetic form in Korea [10–12].Further, CRFs and URFs are widely distributed in countries where different subtypes co-circulate [13–16].

Phylogenetic trees based on viral genes can deliver crucial insights into the ecology and evolution of HIV transmission [17, 18]. Population-level phylogenetic patterns reflect both transmission dynamics and genetic changes [19–21], which accumulate because of selection or drift. Currently, the best method to identify and establish transmission events related to HIV between individuals or within a community is high-resolution phylogenetics based on HIV sequence data [22–26].

In this study, we aimed to determine whether a longitudinally-sampled dataset derived from HIV-1-infected individuals over a 14-year period (1999–2012) could shed light on the transmission processes involved in the initiation of the HIV epidemic in Korea. The identification of transmission clusters and their characterization may provide valuable insights into factors that contributed to the origin of HIV transmission in Korea. We characterized the composition of phylogenetically reconstructed “clusters”, or groups of people in which multiple transmissions likely occurred, and assessed the factors associated with membership to these clusters among patients diagnosed from 1999 to 2012. Here we report our results from applying the phylodynamic profiles of HIV-1 subtype B and other subtypes circulating among the antiretroviral drug-naïve population of Korea.

## Materials and methods

### Study population and RNA extraction

Blood and plasma samples of individuals newly diagnosed with HIV-1 infection, for whom highly active antiretroviral therapy (ART) had not been initiated, were collected on an annual basis for genotypic assays of antiretroviral drug-resistant variants in Korea. Variations in *HIV-1 pol* (a polymerase gene) were monitored continuously using a subset of approximately 10% of the samples isolated from newly-diagnosed drug-naïve patients every year since 1999 (Table 1). A simple random sampling method was used to select patient groups based on their epidemiological history. The study was approved by Korea Centers for Diseases Control and prevention Research Ethics Committee (No. 2012-05CON-11-P).

**Table 1:** Demographic characteristics of the Korean antiretroviral drug-naïve HIV-1-infected population from 1999 to 2012 (n = 927).

Year	1999–2005	2006	2007	2008	2009	2010	2011	2012	2006–2012
Characteristics	(n = 300) ^¶^	(n = 39)	(n = 70)	(n = 118)	(n = 70)	(n = 64)	(n = 125)	(n = 141)	(n = 628)
No. of annual cases ^a^	2,593	749	740	797	768	773	888	868	5,583
**Gender, total n (%)**^b^	300(11.6)	39(5.2)	70(9.5)	118(14.9)	70(9.1)	64(8.3)	125(14.1)	141(16.2)	628(11.4)
Male	279	35	67	109	67	58	116	133	586
Female	21	4	3	9	3	6	9	8	42
**Age (year)**									
Mean	38.3	45.5	40.5	39.8	40.1	41.1	42.4	39.3	41.1
Range	15–71	22–77	17–65	17–68	19–70	2–72	18–75	1–80	1–80
**Subtype**									
B	288	37	67	108	67	61	111	125	576
non-B	12	2	3	11	3	3	14	16	52
**Transmission risk category**									
Heterosexual contact	159	12	42	51	26	26	44	51	252
Homosexual contact	118	18	22	46	24	24	30	36	200
Vertical transmission	-	-	-	-	-	1	-	1	2
Unidentified	23	9	6	22	20	13	51	53	174
**Plasma HIV RNA level**									
Mean log copies/mL	5.42	5	4.87	4.9	4.96	4.85	5.02	4.87	4.92
No. tested	175	39	70	119	70	64	125	141	628
Range	2.45–7.26	3.15–6.32	3.04–6.48	3.09–7.00	3.23–7.00	3.26–6.40	3.18–7.00	3.21–6.58	3.04–7.00

^a^Number of annual cases indicates the total number of cases reported to the Korea Centers for Disease Control and Prevention (KCDC).

^b^Values in parentheses represent the number of tested cases divided by the number of reported cases. Variations in HIV-1 *pol* were monitored continuously using a subset of greater than 10% of samples isolated from newly diagnosed drug-naïve patients every year since 1999 in South Korea.

^**¶**^Ju-yeon Choi et al. J Acquir Immune Defic Syndr. 2008;49: 237–242.

### Viral RNA extraction, *pol* amplification, and sequencing

The experimental conditions for reverse transcription-polymerase chain reaction (RT-PCR) and PCR for sequencing the *PR* and *RT* parts of the *pol* region were based on the laboratory protocol specified by Stanford’s Center for AIDS Research. In accordance with the manufacturer’s instructions, viral RNA was extracted from 140 μL of the plasma sample using a QIAamp Viral RNA Mini kit (Qiagen, Valencia, CA, USA) and was suspended in 50 μL of elution buffer. RT-PCR was used to generate a template with the primer set MAW-26 (2028–2051, 10 pmol/mL, forward) and RT-21 (3539–3509, 10 pmol/mL, reverse), using a SuperScript III One-Step RT-PCR kit and Platinum Taq DNA polymerase (Invitrogen, Carlsbad, CA, USA). After RT-PCR, nested PCR was performed. The PCR product of *pol* (~1.3 kb), containing the entire *PR* and *RT* gene sequences, was subjected to direct sequencing using an ABI Prism Big-Dye Terminator Cycle Sequencing 3.1 Ready Reaction Kit (Applied Biosystems, Foster City, CA, USA) with an automated sequencer (ABI Prism 3730 DNA sequencer; Applied Biosystems), after purification with a Millipore PCR Cleanup kit (Millipore Corp., Madison, WI, USA) and following gel electrophoresis.

### Genotypic drug resistance assays

*PR* and *RT* drug resistance mutations in antiretroviral drug-naïve patients were investigated to analyze genotypic resistance to protease inhibitors (PIs) and nucleoside reverse transcriptase inhibitors (NRTIs), as well as non-NRTI (NNRTI)-related resistant variants. Resistant mutations were identified based on the consensus statement from the Stanford sequence database (DB) for HIV *PR* (codons 1–99) and *RT* (codons 1–300). The defined drug resistance positions were as follows: 22 protease inhibitor-resistance positions at codons 10, 20, 24, 30, 32, 33, 36, 46, 47, 48, 50, 53, 54, 63, 71, 73, 77, 82, 84, 88, 90, and 93; 18 NRTI-resistance positions at 41, 44, 62, 65, 67, 69, 70, 74, 75, 77, 115, 116, 118, 151, 184, 210, 215, and 219; 15 NNRTI-resistance positions at 98, 100, 101, 103, 106, 108, 179, 181, 188, 190, 225, 227, 230, 236, and 238. In addition, the Stanford HIVdb (Drug Resistance Algorithm, Beta Test [version 2004.04]) was used to calculate the level of resistance against each antiretroviral drug (release notes for HIVseq, HIVdb, and HIValg: http://hivdb.stanford.edu/). The intensity of drug resistance was classified as S (susceptible, potential low-level resistance), I (low-level, intermediate-level resistance), or R (high-level resistance) on the guidelines specified by the Stanford DB.

### HIV genotyping and phylogenetic analysis

HIV-1 subtype and CRF designations were determined by uploading the sequences into REGA HIV-1 automated Subtyping Tool version 2.0 (http://www.bioafrica.net/regagenotype /html/subtypinghiv.html) and were confirmed by in-house phylogenetic analysis of *pol* nucleotide sequences, as described previously [3, 4, 17]. For phylogenetic analysis, reference sequences representing the overall genetic variability of HIV-1 group M, including all subtype, sub-subtype, and CRF references, were obtained from the National Institutes of Health/National Institute of Allergy and Infectious Diseases (NIH/NIAID)-funded HIV database. Phylogenetic analysis was performed using MEGA 6.06 initially, and an alignment of 1437 nucleotides was created. Genetic subtype and potential transmission clusters from the time-stamped sequence dataset were first deduced by neighbor-joining tree reconstruction using MEGA version 6.06 based on the GTR G+I parameter model [27]. The robustness of the transmission clusters was further tested by the more rigorous maximum likelihood inference implemented by MEGA version 6.06 [27] using a gamma distribution with discrete gamma categories. The reliability of the branching orders was assessed by bootstrap analysis of 1000 replicates. The most appropriate nucleotide substitution model was determined using FindModel, a web implementation of Modeltest available at the HIV DB (http://www.lanl.gov.com). Using the Bayesian Markov Chain Monte Carlo framework, 100 million steps were performed, sampling every 10,000, and removing 10% as burn-in. Convergence was assessed using Tracer (v1.4), and effective sample size values greater than 200 were accepted. A maximum clade credibility tree was summarized with TreeAnnotator (available in the BEAST package) and was visualized with Figtree (v1.4) [28, 29].

### Identification of transmission clusters and analysis of associations with transmission clustering

Phylogenies were inferred using a general-time reversible model of nucleotide substitution, an estimated proportion of invariant sites, and gamma distributed rates among sites. The best sub-tree pruning, and re-grafting and nearest neighbor interchange heuristic options were selected to search the tree space, and bootstrap values with 1000 replicates were used to assess confidence in topology. The existence of transmission clusters was determined based on the statistical robustness of the maximum likelihood topologies assessed by high bootstrap values (90%) with 1000 re-samplings and short branch lengths (genetic distances, 0.015) of the HIV-1 *pol* sequence. The phylogenetic tree was displayed with FigTree v.1.42 (tree.bio.ed.ac.uk/software/figtree/).

We considered membership in a phylogenetic transmission cluster as the dependent variable for our analysis. We used a Pearson’s chi-squared test, a Fisher’s exact test, and Student *t*-tests to compare clustered and non-clustered patient samples. We then entered variables with a *P* value of 0.2 based on preliminary analysis or variables that we considered conceptually important based on previous reports, into univariable and multivariable regression models to examine associations with transmission clustering [16]. We conducted multivariable analysis using the full data set. The analysis was then repeated using samples with available transmission risk data. Among men with a known risk of transmission, we further stratified the regression analysis to identify differences in associations based on clustering comparing men who had sex with men (MSM) versus men who had not had sex with men (non-MSM). We based the multivariable model building on an iterative approach and assessed model fit using the Hosmer-Lemeshow goodness-of-fit test. We analyzed data using SAS version 9.2 (SAS Institute, Cary, NC, USA).

## Results

### Patient demographics and molecular characteristics of HIV-1

In total, 927 sequences were acquired from 1999–2012, with each sequence acquired from an individual patient who had not received treatment. Seventy-four percent of the population was greater than 30 years of age (median age, 34.5 years). Men comprised 89.4% (829/917) of the study population, whereas women accounted for 6.6% (61/917) of the total HIV-1-infected population. This ratio was similar to that reported by the Division of HIV and TB Control, KCDC for the entire HIV/acquired immunodeficiency syndrome (AIDS) population in Korea in March 2013 (90.9% men and 9.1% women). The majority of men (324; 39.1%) attributed their infection to heterosexual contact; 285 (34.4%) were classified as MSM, and two (0.2%) infections were perinatal. The women attributed their infections to heterosexual contact. Table 1 shows the demographic characteristics of the 927 patients. The samples were characterized according to sex, age, transmission route, CD4 count, and HIV RNA levels in the plasma.

### HIV-1 genotyping and URF identification

Of the 927 samples, 863 (93.1%) were classified as subtype B and 64 were classified as other subtypes (non-B; 6.9%; Table 1). The non-B group consisted of subtypes CRF01_AE (28 isolates, 2.3%), CRF02_AG (12 isolates, 0.6%), G (five isolates, 0.5%), C (five isolates, 0.5%), CRF06_cpx (five isolates, 0.5%), A (three isolates, 0.3%), CRF07_B/C (one isolate, 0.1%), CRF56_cpx (one isolate, 0.4%), and several URFs (five isolates). Bootscan analysis (REGA 3.0) revealed that these URFs possessed breakpoints that differed from any known reference sequences (Table 2). Recombinants CRF06/B or B/G URFs (five samples) possessed a distinct mix of subtype B and other subtypes. Further full genome sequencing needs to be performed to confirm the presence of novel circulating recombinants in these samples.

**Table 2:** Characteristics of the HIV-1 subtypes in Korea from 1999 to 2012.

Subtypes	1999	2000	2001	2002	2003	2004	2005	2006	2007	2008	2009	2010	2011	2012	Total
HIV-1 Subtype B	34	20	36	33	46	73	37	37	66	107	68	61	113	126	857
HIV-1 CRF 01_AE		2	1	3	1	2				6	1	1	5	6	28
HIV-1 CRF 02_AG		1				1			1	2	1	2	1	3	12
HIV-1 CRF 06_CPX						1	1	1						1	4
HIV-1 CRF 07_BC													1		1
HIV-1 Subtype A (A1)						1							1	1	3
HIV-1 Subtype C						1				2			2		5
HIV-1 Subtype G						1			1				1	2	5
HIV-1 CRF 56_CPX													1		1
Recombinant of 06_CPX, B								1							1
Recombinant of 01_AE, B		1													1
Recombinant of A1, B		1													1
Recombinant of B, D						1			1	1				2	5
Recombinant of B, F1									1						1
Recombinant of B, G		1													1
Recombinant of G, B		1													1
Total	34	27	37	36	47	81	38	39	70	118	70	64	125	141	927

### Identification of the transmission clusters

**Fig 1:**
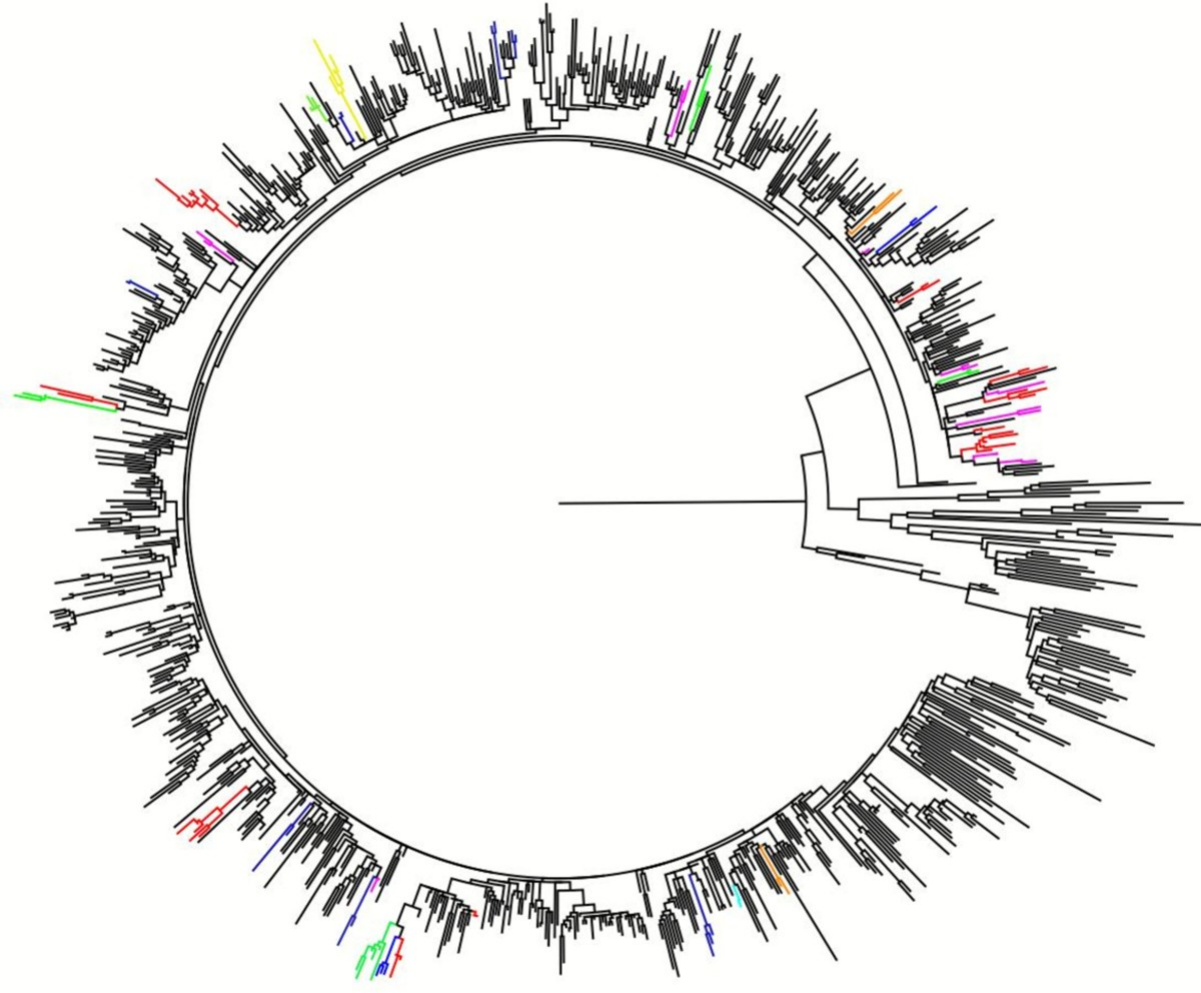
Phylogenetic clustering and genotyping of samples from HIV patients diagnosed between 1999 and 2012. Phylogenetic tree depicting newly diagnosed patients with HIV in Korea between 1999 and 2012. Of the 927 patients, 104 patients are shown here based on 37 transmission clusters, grouped by color, and labeled as clusters 1–37, with each tip representing an individual patient. Each depicted cluster satisfied greater than 90% support based on the bootstrap resampling cut-off. The scale line indicates genetic distance.

Based on viral sequences, phylogenetic analysis demonstrated that 104 of 927 patients (11.2%) were grouped into 37 clusters. All clusters were composed of 2–8 members; for seven small clusters, both or all three patients that formed the clusters were diagnosed during the same year. Twenty-two clusters included individuals who first consulted a hospital 2–4 years after the report of infection; the remaining eight were clusters that included patients for whom presentation covered 5 years or more prior to consulting a hospital. The mean number of patients per cluster was 2.81 (range, 2–8). Interestingly, all clusters belonged to subtype B, and four large clusters (comprising six or eight patients) and 33 smaller clusters were identified. Large cluster no. 31 comprised young men (mean age, 22.3 years), who exhibited a slightly lower viral load than patients in the other clusters and accounted for a long time span (2004–2012). Phylogenetic trees based on the viral sequences of the clustered and non-clustered patients are shown in Fig 1. An overview of the characteristics of the clustered and non-clustered patients is given in Table 3. Being part of a transmission cluster was significantly associated with harboring a subtype-B virus, infection through sexual contact, male sex, and being a young male. The characteristics of the patients in part of the transmission clusters are summarized in Table 4.

**Table 3:** Factors associated with cluster membership: full sample analysis (n = 927).

		Total	In a Cluster, n	Not in a Cluster, n	Adjusted Odds Ratio (CI)
Total		927	104	823	
Age					
	Mean	40.2	38.2	40.5	
	≤ 30	216	36	180	1
	> 30	675	66	609	1.875 (1.200, 2.929)
Sex					
	Male	829	99	730	1
	Female	99	5	94	2.915 (0.889, 9.559)
Risk factor data					
	Yes	674	82	592	1
	No	254	22	232	1.400 (0.832, 2.357)
CD4 count, cell/mm^3^, n					
	< 200	643	66	577	1
	200–349	132	16	116	1.007 (0.554, 1.833)
	350–499	77	10	67	1.106 (0.535, 2.289)
	≥ 500	76	12	64	1.415 (0.713, 2.809)
HIV transmission risk					
Male		611	78	533	
	MSM	286	41	245	
	Heterosexual	323	37	286	
	Perinatal	2	0	2	
Female		42	3	39	
	Heterosexual	42	3	39	
	Perinatal	0	0	0	
HIV RNA, log (copies/mL)		764	95	669	
	Mean	11.3	11.3	11.3	

**Table 4:** Demographic and clinical characteristics of the transmission clusters in this study.

Cluster ID	Code	CD4	RNA (copies/ml)	Age	Sex	Sexual behavior	Drug Resistance sites	Genetic Distance	Bootstrap score
Cluster 19	2080360	228	119,801	40	M	Heterosexual		0.027	0.982
	2100068		176,288	46	M	MSM			
	2110230		169,307	44	M	Unknown			
	2120309	141	198,621	75	M	MSM	Y181SY, NNRTI(NVP;R)		
	2120639		322,739	70	M	Unknown			
	2110450	91	665,353	49	M	MSM			
	2120240		8,670	48	M	MSM			
	2120517		1,116,776	36	M	Unknown			
Cluster 13	2090700		47,926	45	M	Heterosexual		0.028	0.997
	2100535	395	23,108	41	M	MSM			
	2100451	342	1,068,032	32	M	MSM			
	2100514	666	25,897	48	M	Unknown			
	2110191	63	1,134,644	27	M	MSM			
	2110359	585	3,520	31	M	Heterosexual			
Cluster 23	2090148	510	286,382	31	M	Heterosexual	E138G, NNRTI (RPV;I)	0.026	0.955
	2100204	704	7,699	31	M	Unknown	E138G, NNRTI (RPV;I)		
	2120031	328	248,115	39	M	Heterosexual	E138G, NNRTI (RPV;I)		
	2120612		81,561	37	M	MSM	E138G, NNRTI (RPV;I)		
	2120696	617	49,302	41	M	Heterosexual	E138G, NNRTI (RPV;I)		
	2120591	474	59,873	25	M	MSM	E138G, NNRTI (RPV;I)		
Cluster 31	204066		13,000	20	M	MSM		0.029	0.907
	2090102	272	14,743	23	M	Heterosexual			
	2090210	92	62,937	24	M	Unknown			
	2110441		10,245	20	M	Unknown			
	2120279	533	239,733	25	M	Heterosexual			
	2120614	237	22,602	22	M	Heterosexual			
Cluster 4	204059		960	25	M	Heterosexual		0.027	0.997
	206165		230,000	43	M	Heterosexual			
	2070086	62	520,000	51	M	Heterosexual			
	2080746	103	157,193	40	M	Heterosexual			
Cluster 5	206445		1,600,000	40	M	Unknown		0.011	0.933
	2080108		2,500	68	M	MSM			
	2080534	411	640,319	55	M	Heterosexual			
	2110863		1,528,944	47	M	Heterosexual			
Cluster 11	210495		17,439	46	M	MSM	K101E, E138A, NNRTI (EFV, ETR, NVP, RPV)	0.015	1
	2100542		30,213	31	M	Heterosexual	K101E, E138A, NNRTI (EFV, ETR, NVP, RPV)		
	2120209		1,920	25	M	Unknown	K101E, E138A, NNRTI (EFV, ETR, NVP, RPV)		

### Transmission clusters related to transmitted drug resistance

We further investigated whether transmission of drug-resistant strains occurred among these 37 clusters. Of all the clusters, three (~8.1%) that included 10 individuals (22.2% of 45 individuals) included at least one member with transmitted drug resistance (TDR). Of these, in two clusters, all members had TDR. The largest cluster with TDR (cluster no. 23) had an E138G mutation related to NNRTI (RPV), and cluster no. 11 had K101E and E138A mutations related to NNRTI (EFV, ETR, NVP, and RPV; Table 3). The homogenous TDR cluster no. 11 had a higher bootstrap score (mean, 1.00 versus 0.97) and mean genetic distance (0.015 substitutions/site) compared to those in clusters that had no members with TDR. TDR cluster no. 23 had a lower bootstrap score (mean, 0.95 versus 0.97) and a higher mean genetic distance (0.026 substitutions/site) compared to those in clusters that had no members with TDR.

### Factors associated with membership in the phylogenetic cluster

Based on the initial exploratory analysis, the clustered patients were younger (median age, 36.5 versus 39.0 years) and were more likely to be male (98.0%) for all comparisons (Table 1). Clustered patients frequently had CD4 counts greater than 350 cells/mm^3^. Based on multivariable analysis, factors of less than 30 years of age, male gender, and CD4 counts greater than 350 cells/mm^3^ were found to be associated with cluster membership (Table 2). Clinical records revealed the HIV transmission risk factor data for 674 of 927 (72.7%) individuals; however, no differences in risk factor data availability were detected between clustered and non-clustered patients. Among the men with available risk factor data, MSM transmission risk was slightly more common among clustered patients (Table 5). Our additional multivariable analysis, which included a subgroup for which HIV transmission risk data was available, revealed an association between clustering and age less than 30 years, male gender, MSM status, and higher CD4 counts (Table 5).

**Table 5:** Factors associated with cluster membership: MSM only.

		Total	In a Cluster, n	Not in a Cluster, n	Adjusted Odds Ratio (CI)
Total		300	42	258	
Age					
	Mean	37.9	38	37.9	
	< 30	86	17	69	1
	> 30	201	24	177	2.010 (0.995, 4.060)
CD4 count, cell/mm^3^, n					
	< 200	33	3	30	1
	200–349	29	6	23	0.738 (0.293, 1.859)
	350–499	54	7	47	1.478 (0.542, 4.029)
	≥ 500	184	26	158	0.490 (0.136, 1.764)
HIV RNA, log (copies/mL)		235	36	199	
	Mean	11.1	11.4	11.1	

### Characteristics of the MSM population

Information on the transmission route was available for 674 of 927 patients. Of these, 300 (44.1%) reported transmission through homosexual contact with men (MSM), and 367 (54.5%) reported transmission through heterosexual contact. Phylogenetic analysis demonstrated that 42 of 300 MSM (14%) grouped into clusters. The clustered patients were more likely to have CD4 counts greater than 350 cells/mm^3^. Based on multivariable analysis, age less than 30 years, male gender, and CD4 counts greater than 350 cells/mm^3^ were associated with cluster membership (Table 5). Samples from all MSM clusters were grouped as subtype B. Interestingly, the other clusters were grouped into the monophyletic subtype B group, which includes the Korean clade B, based on *Env* sequences [30, 31].

## Discussion

In this study, we compared HIV *pol* sequences from 927 newly-diagnosed patients receiving care at the Division of AIDS, KNIH between 1999 and 2012. We found that 11.2% of these patients grouped into 37 molecularly-defined HIV transmission clusters. We analyzed the structures of the transmission clusters in Korea through the integration of molecular, clinical, and demographic data. Analysis of the HIV-1 *pol* sequences generated from antiretroviral resistance surveillance programs has proven useful and informative to assess and define transmission clusters within a population of interest [4, 15, 32]. Based on these criteria, substantial clustering (11.2%, 104/927) was observed in the current study, indicating that the majority of HIV-1 subtype B infections in Korea were linked to a cluster that might be associated with local and/or foreign HIV-1 networks.

Real-time identification of epidemiological hot spots, pinpointing viral transmission chains and biologically linking the drivers of the epidemic, is necessary to introduce effective prevention strategies and interrupt HIV transmission chains [3, 24, 33]. Identifying clusters that account for the largest proportion of onward transmissions and characterizing the structural, behavioral, and biological correlates that predict transmissibility will aid in the development of targeted interventions [33, 34]. The events listed here represent the first identified transmission events of HIV among the general population in Korea.

Surprisingly, subtype B remained the dominant circulating subtype, contributing to approximately 92.5% of all cases of HIV-1 infection in Korea. This finding is in contrast to the results reported for the neighboring countries of China and Japan [2, 25, 35, 36]. In the present study, a number of recombinants involving subtype B and other types were observed. Interestingly, the subtype-B region of these URFs in the Korean MSM population was found to be of western B origin, distinguishing them from the URFs recently described among people who were infected with subtype B of Chinese or Japanese origin [26, 37–39]. The emergence of these variants indicates an ongoing active intersubtype recombination event between the predominant genotypes. Such events might complicate disease management.

In this study, we found that the presence of TDR, in addition to young age and male sex, was significantly associated with transmission cluster membership. In particular, in the multivariable model, the association between TDR and clustering remained significant, even after controlling for infection duration. These associations with clustering might simply be markers of very high-risk behaviors and rapid ongoing transmission, as there is no evidence to suggest that TDR mutations make the virus more transmissible [14, 22, 40]. Furthermore, we found several clusters in which nearly all individuals harbored TDR, indicating that drug-naïve individuals contribute to the onward spread of TDR. Importantly, these clusters might reflect sexual networks that are reservoirs of drug resistance beyond ART-experienced individuals.

The incidence of HIV remains low in Korea compared to that in many other countries. In 2013, there was a cumulative total of 10,404 HIV/AIDS cases in Korea, of which approximately 80% were associated with sexual contact, based on available data (KCDC reports, 2013). The number of newly reported HIV cases has increased by more than 5-fold during the past decade, from 199 in 1999 to 1114 in 2013. Despite extensive HIV prevention and education programs targeting at-risk groups, many MSM individuals in Japan appear to engage in high-risk sexual behavior, predisposing them to HIV-1 infection and transmission. Based on our subgroup analysis examining the risk factors associated with HIV transmission clustering among men, the finding that being young and male was only significantly correlated with clustering for non-MSM individuals (versus MSM) might have resulted from the fact that both the clustered and non-clustered MSM groups included a significantly high number of individuals who were less than 30 years of age.

Interestingly, other studies conducted in Asia (China, Japan, and Malaysia) reported similar findings during the same period, indicating that the emergence of the transmission clusters was likely caused by increased exposure to high-risk behaviors among MSM individuals [23, 41–43]. HIV-1 epidemics among MSM groups in major cities in the United States and Europe were first detected in the early 1980s; in these epidemics, subtype B was the founder strain responsible for these events [44]. Subtype B is also commonly found in most developed countries of Western Europe, as well as in South American and Asian countries [6, 18, 39, 45, 46]. According to Kim et al., who studied samples collected between February 1998 and March 2005 in Korea, subtype B was virtually the only HIV-1 strain identified among MSM individuals in Korea [31].

From a public health perspective, sexual networks within the MSM population could serve as an important force of continual HIV-1 dissemination, thereby representing key entry points for the delivery of intervention strategies. A previous study revealed a significant association between high-risk sexual behavior and ignorance regarding HIV infection [4].

## Conclusion

Analysis of the phylodynamic or evolutionary history of HIV relies significantly on the depth of population-based sampling; a study of this type should be continued and expanded upon to improve the resolution of HIV-1 genomic diversity and transmission dynamics within HIV transmission networks. The continuing transmission of HIV among MSM individuals indicates the need to maximize the use of available bio-epidemiological data. HIV transmission cluster analysis integrates laboratory data with behavioral data to enable the delineation of key transmission patterns to develop tailored interventions aimed at interrupting transmission chains.
